# Influence of a Static Magnetic Field on the ⟨100⟩ Growth Rates of Sodium Chlorate Crystals from Aqueous Solution

**DOI:** 10.1021/acsomega.2c04790

**Published:** 2022-12-09

**Authors:** Milica M. Milojević, Branislava M. Vučetić, Biljana Z. Maksimović, Olivera R. Klisurić, Mićo M. Mitrović, Andrijana A. Žekić

**Affiliations:** † Faculty of Physics, University of Belgrade, Studentski trg 12, 11000Belgrade, Serbia; ‡ Faculty of Sciences, Department of Physics, University of Novi Sad, Trg Dositeja Obradovića 4, 21000Novi Sad, Serbia

## Abstract

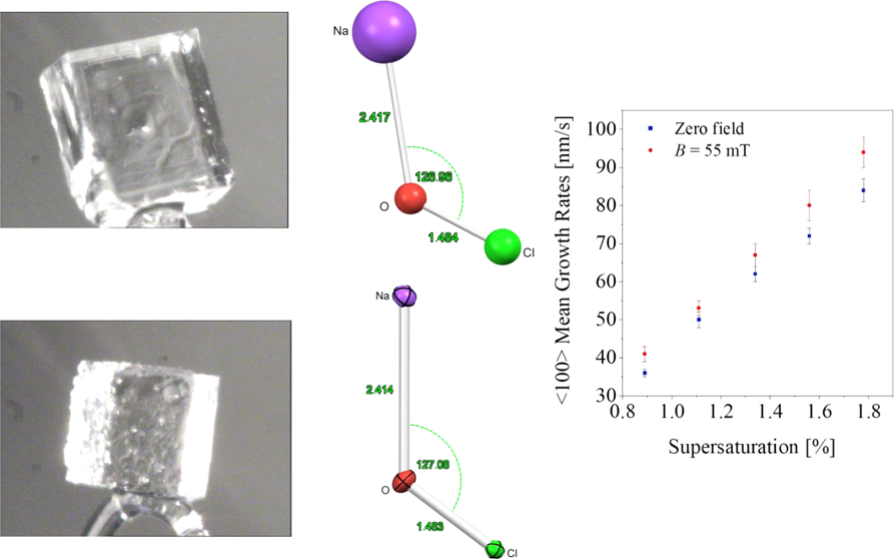

The results of the study of the influence of a static magnetic field of 55 ± 3 mT on the growth rates of diamagnetic sodium chlorate crystals in the direction ⟨100⟩ will be presented. Two groups of experiments were performed in the same solution supersaturation range of 0.89–1.78%, the first in zero field conditions, and the second in an applied magnetic field. The results show that crystals nucleated and grown in a static magnetic field have higher mean growth rates in the ⟨100⟩ direction than crystals in a zero field. Also, X-ray analyses suggest that crystals nucleated and grown in a magnetic field may have a higher lattice constant. Possible mechanisms and possible reasons for these phenomena are discussed.

## Introduction

Although the effect of external magnetic field on the growth of crystals from solution has been the focus of many studies for decades, it still attracts the attention of researchers. The results of previous research show that the external magnetic field can increase or decrease the growth rates of the studied crystals, but it can also have no effect on their growth. It has also been shown that the magnetic field affects the growth of para- and diamagnetic crystals differently. Sometimes, studying the influence of the magnetic field on the growth rates of the same type of crystal leads to opposite results. These are just some of the reasons to continue research in this field.

Half a century ago, Schieber^1^ studied the isothermal growth/dissolution of paramagnetic Fe(NH_4_)_2_(SO_4_)_2_·6H_2_O and diamagnetic KAl(SO_4_)_2_·12H_2_O crystals, in a homogeneous magnetic field up to 140 kOe. The results showed that an external magnetic field increased both growth/dissolution rates of paramagnetic crystals, while no changes in rates of diamagnetic samples occurred.^1^ Kuschel et al.^2^ have shown that magnetic field *B* ≤ 1.4 T did not affect the normal growth rate of diamagnetic Zn(NH_4_)_2_(SO_4_)_2_·6H_2_O or paramagnetic Cu(NH_4_)_2_(SO_4_)_2_·6H_2_O and Fe(NH_4_)_2_(SO_4_)_2_·6H_2_O crystals. The growth rates of paramagnetic Ni(NH_4_)_2_(SO_4_)_2_·6H_2_O crystals increased slightly at high values of solution supersaturation. The same authors showed that the (110) face growth rate of paramagnetic Co(NH_4_)_2_(SO_4_)_2_·6H_2_O, parallel to the magnetic field up to 7 T, exhibits a small increase.^3^ This effect increased with increasing supersaturation of the solution. On the other hand, the {110} face growth rates of diamagnetic Ni(NH_4_)_2_(SO_4_)_2_·6H_2_O, as well as {100} face growth rates of diamagnetic sodium chlorate crystals showed no changes during growth in a magnetic field, at supersaturation of 2 and 11%, respectively.^3^

The growth rates of cadmium phosphate crystals nucleated and grown at different supersaturations were higher for crystals grown in a magnetic field of 0.27 T, than for crystals growing in zero field.^4^ The displacement rate of the (100) faces of ammonium dihydrogen phosphate crystals was lower when a magnetic field of 0.17 T was applied.^5^ Also, a magnetic field of 0.22 T decreases the growth rates in the [010] direction of most Rochelle salt crystals studied^6^ and of most MnCl_2_·4H_2_O crystals in the direction perpendicular to the plane (100).^7^ A magnetic field of 0.18 T also decreases the growth rates of calcite.^8^

Schieber^1^ proposed four possible mechanisms to explain the influence of the magnetic field on the growth of crystals from solution, such as the thermodynamic effect, magnetohydrodynamic effect, magnetic dipolar interaction, and gradient of the magnetic field. Kuznetsov et al.^9^ have proposed the wave mechanism of the effect of external fields on crystallization.

In this paper, the results of the study of the influence of the magnetic field on the ⟨100⟩ growth rates of diamagnetic sodium chlorate crystals, in a certain range of the solution supersaturation are presented. Also, the results of the study on the possible influence of the applied field on the parameters of the crystal lattice are presented.

## Experimental Section

The main objective of the research was to determine the possible influence of the static magnetic field on the growth rates of small sodium chlorate crystals in the ⟨100⟩ direction in isothermal experiments. The relative supersaturation of the solution was calculated as σ = (*c* – *c*_0_)/*c*_0_, where *c* is the actual solution concentration and *c*_0_ is the saturated solution concentration. Concentrations were calculated using the empirical formula *c* = 0.226*t* + 44.38 (g NaClO_3_/100 g solution),^10^ where *t* is the temperature of the solution. Solutions were prepared by dissolving sodium chlorate of 99% purity in deionized water and then maintained at the saturation temperature *T*_0_ for 3 days, prior to experiments.

**Figure 1:**
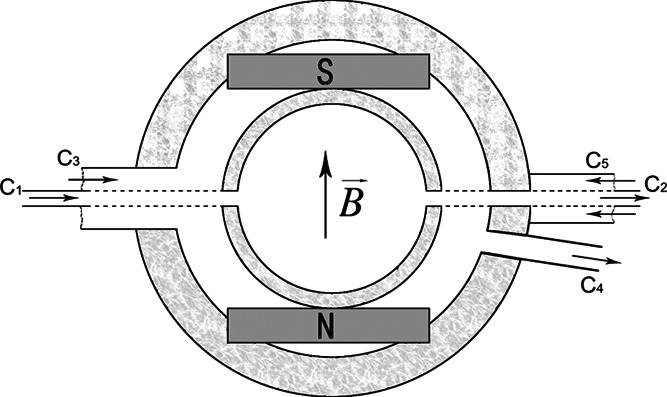
Schematic drawing of crystallization cell (top view). C_1_: solution inlet pipe; C_2_: solution outlet pipe; C_3_, C_4_, C_5_: thermostatically controlled water lines; S, N: magnetic poles.

The experimental setup used for the research is described in detail elsewhere.^11^ Nucleation and crystal growth took place in a cell that, for the purposes of these experiments, consisted only of plastic and glass components. The diameter of the cell was 36 mm, the height was 15 mm, and the volume was 15 mL. The flow rate of the solution through the cell was about 0.5 mL s^–1^, while the velocity of the solution around the crystals at the bottom of the cell was about 0.05 mm s^–1^. The temperature of the solution in the cell was kept constant within ±0.02 °C. Perpendicular to the solution flow, two neodymium magnets can be attached to the opposite sides of the cell. This allows experiments in two modes, with and without a magnetic field. The magnets generate a static magnetic field of 55 ± 3 mT, measured with an AC/DC magnetic field meter, Datalogger SDL900, EXTECH Instruments, in a nearly square space with dimensions 16 × 16 mm^2^, inside the cell. Figure 1 shows a schematic drawing of a crystallization cell for crystal growth in a magnetic field.

**Figure 2:**
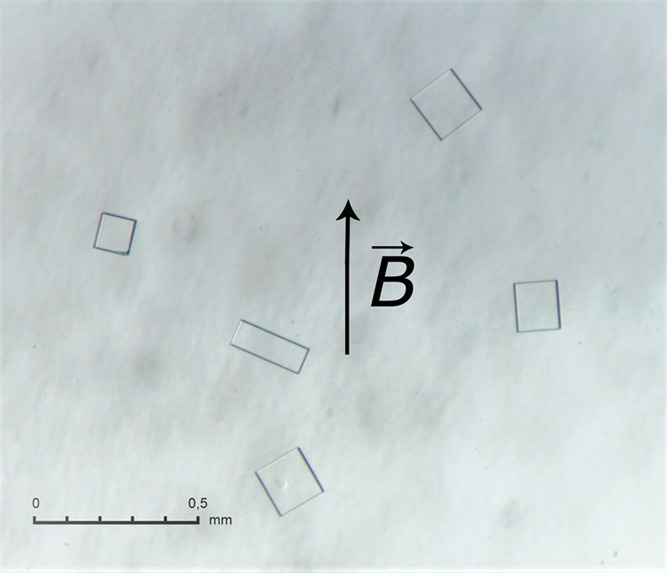
Crystal arrangement in the part of the cell with the magnetic field of 55 ± 3 mT.

To determine the influence of the magnetic field on the ⟨100⟩ growth rates of the observed crystals, two groups of experiments were carried out. The first group includes growth runs in which sodium chlorate crystals were nucleated and grew in a zero magnetic field. The second group includes growth runs in which crystals were nucleated and grown in the part of the cell exposed to a magnetic field of 55 ± 3 mT. All observed crystals were spontaneously nucleated at a temperature of *T* = 28.0 ± 0.1 °C. After nucleation, the crystals grew at the same temperature for about 4 h. The arrangement of the crystals after nucleation in the part of the cell exposed to a magnetic field of 55 ± 3 mT is shown in Figure 2.

To observe crystal growth a transmitted light microscope, Nikon SMZ800, equipped with Luminera camera, Infinity 1, was used. During the first 45 min of their growth, the crystals were photographed every 15 min, and then every 30 min until the end of the growth run. Crystal length in the ⟨100⟩ direction was measured with an accuracy of about ±5 μm using Infinity Analyzer software. To determine the average growth rates of the observed crystals, the least-squares method was applied to crystal length vs time dependence. Average growth rates with measurement errors less than 3% were included in further analyses. The Origin Pro 2022 software package was used to determine the average and mean growth rates of observed crystals.

To determine the possible influence of the external magnetic field on the lattice parameters, X-ray diffraction was performed on selected single crystals grown under the same solution supersaturation, in the zero field conditions, and in the applied magnetic field. Suitable single crystals were mounted on an optical fiber and crystallographic data were collected using a Rigaku (Oxford Diffraction) Gemini S diffractometer with a CCD area detector and incident monochromatic graphite wavelength radiation λ_Mo Kα_ = 0.71073 Å at 293 K. The CrysAlis Pro and CrysAlis Red software packages^12^ were used for data acquisition and integration. The collected data were corrected for absorption effects using a multiscan absorption correction.^13^ The crystal structures were solved using the SHELXT^14^ algorithm for intrinsic phase determination implemented in the OLEX2^15^ graphical user interface. The structure was subsequently refined using SHELXL-2018/3.^16^ Atoms were freely refined with anisotropic displacement parameters.

## Experimental Results

All observed crystals, pertaining to both groups of experiments, in zero field conditions and in the applied magnetic field of 55 ± 3 mT, grew under the same conditions such as growth temperature, supersaturation, and hydrodynamics of the solution. Table 1 presents the growth conditions: *T*_0_ is the saturation temperature, σ is the corresponding supersaturation of the solution, as well as the obtained experimental results; *N*_z_ and *N*_f_ are the total number of ⟨100⟩ growth rates in the zero field and in the applied magnetic field, respectively; *R̅*_z_ and *R̅*_f_ are the ⟨100⟩ mean growth rates in the zero field and in the applied magnetic field, respectively; and δ_*R̅*_ is the relative change in the ⟨100⟩ mean growth rates.

**Table 1:** Experimental Conditions and Results

*T*_0_ [°C]	σ [%]	*N*_z_	*N*_f_	*R̅*_z_[nm/s]	*R̅*_f_[nm/s]	δ_*R̅*_ [%]
30.0	0.89	220	208	36 ± 1	41 ± 2	14.2
30.5	1.11	151	176	50 ± 2	53 ± 2	7.7
31.0	1.34	185	172	62 ± 2	67 ± 3	8.7
31.5	1.56	182	164	72 ± 2	80 ± 4	10.9
32.0	1.78	185	192	84 ± 3	94 ± 4	12.6

**Figure 3:**
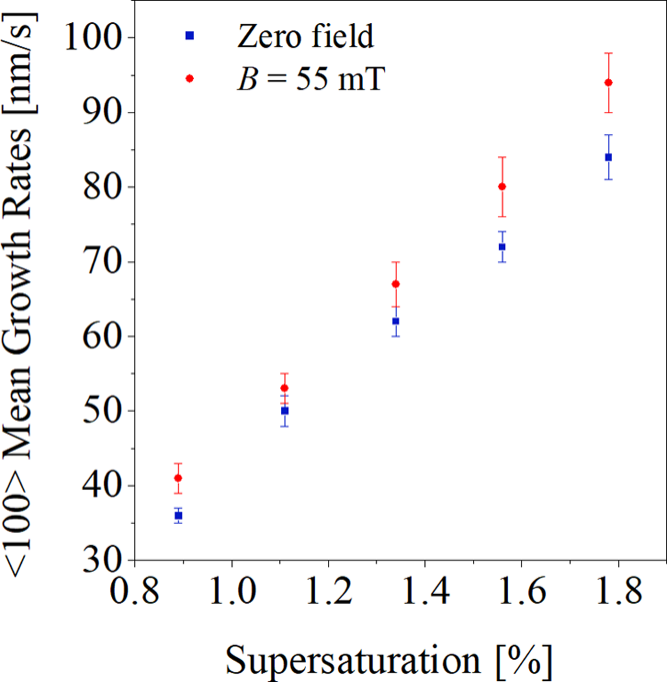
Mean growth rates vs solution supersaturation for zero field conditions (blue squares) and applied magnetic field (red dots).

Figure 3 shows the dependence of the ⟨100⟩ mean growth rates on relative solution supersaturation for crystals observed in the zero field and for crystals observed in the external magnetic field.

**Figure 4:**
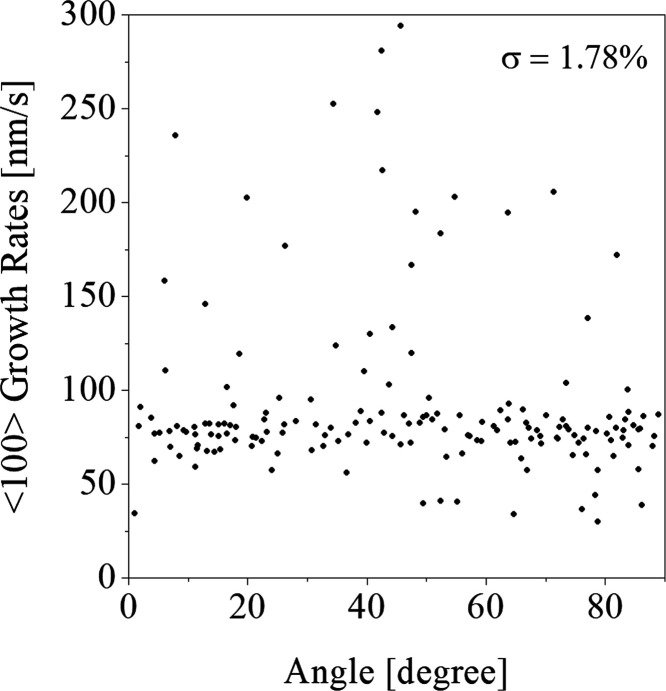
Growth rates vs angle between the magnetic field and the ⟨100⟩ direction.

Figure 4 illustrates the dependence of the growth rates of the crystals in the direction ⟨100⟩ on the angle between the magnetic field and the crystal direction ⟨100⟩, for crystals grown at a relative supersaturation of σ = 1.78%. Similar dependencies were obtained for other supersaturations used.

Since it was noted that several crystals oriented 40–60° to the magnetic field grew at the highest rates, an additional analysis was performed. The growth rates of crystals grown in the zero field conditions and in the applied magnetic field, obtained for all studied supersaturations, were classified into groups according to the angles between the ⟨100⟩ and the field directions. Between 23 and 56 growth rates were in each of the groups. Table 2 shows the relative changes in mean growth rates in the ⟨100⟩ direction, of differentiated groups, due to the magnetic field.

**Table 2:** Relative Changes in the ⟨100⟩ Mean Growth Rates of Differentiated Growth Rate Groups

	relative changes in growth rates [%]					
angle [degree]	σ [%]					average
	0.89	1.11	1.34	1.56	1.78	
0–20	12.7	9.1	–7.5	14.6	14.0	8.6
20–40	7.4	–3.0	13.4	16.2	9.1	8.6
40–60	25.7	6.3	10.3	39.8	30.9	22.6
60–80	14.3	7.0	14.9	–10.8	12.6	7.6
80–90	–21.3	3.1	35.5	–14.7	–4.8	–0.4

A significant difference in the relative changes of mean growth rates can be occurred in directions ⟨100⟩ parallel and orthogonal to the magnetic field. Even in four of five supersaturations used, they have opposite signs. This is in accordance with the predicted anisotropy of crystal growth rates in orthogonal directions in the magnetic field.^9^

Therefore, the additional experiments were designed with an appropriate orientation of the crystals with respect to the field. Initially, nucleation of the crystals occurred at a relative supersaturation of σ = 1.78%. After a certain growth time of about 2 h, the crystals were removed from the cell. For ease of handling, larger crystals, which grew at higher rates in both directions, were selected. These crystals were attached to the glass substrate so that one ⟨100⟩ direction was parallel to the direction of the magnetic field and then reinserted into the cell to continue growing for about 2 h at the same supersaturation as in the first part of the experiments. Table 3 lists the mean growth rates of the mutually orthogonal ⟨100⟩ directions in the first part of the experiments, *R̅*_1_ and *R̅*_2_ respectively; the ⟨100⟩ mean growth rates parallel and orthogonal to the direction of the applied field in the second part of the experiments, *R̅*_1_^′^ and *R̅*_2_^′^, respectively; and the relative changes in growth rates parallel, δ_*R̅*_, and orthogonal to the magnetic field, δ_*R̅*_^′^.

**Table 3:** Parameters Describing Crystal Growth in Mutually Orthogonal ⟨100⟩ Directions, Parallel, and Orthogonal to the Magnetic Field

	*R̅*_1_[nm/s]	*R̅*_1_^′^[nm/s]	*R̅*_2_[nm/s]	*R̅*_2_^′^[nm/s]	δ_*R̅*_ [%]	δ_*R̅*_^′^ [%]
1	102 ± 3	94 ± 1	106 ± 2	93 ± 2	4.7	–0.5
2	96 ± 2	92 ± 1	108 ± 2	104 ± 2	12.3	12.1
3	92 ± 1	105 ± 2	103 ± 3	92 ± 1	12.2	–12.0
4	81 ± 1	109 ± 2	97 ± 2	107 ± 3	19.5	–1.9
5	91 ± 1	90 ± 1	85 ± 1	106 ± 3	–6.7	17.3
6	94 ± 2	88 ± 1	86 ± 2	98 ± 2	–9.1	10.5
7	109 ± 3	92 ± 2	85 ± 1	121 ± 3	–21.8	32.3
8	105 ± 2	93 ± 2	106 ± 3	102 ± 2	0.8	9.3
9	108 ± 3	98 ± 3	81 ± 2	122 ± 3	–25.0	24.4
10	100 ± 2	121 ± 3	91 ± 2	97 ± 1	–8.8	–19.8
11	100 ± 2	104 ± 2	64 ± 1	98 ± 2	–36.2	–5.4
12	92 ± 1	103 ± 2	113 ± 2	84 ± 2	22.3	–18.0
13	91 ± 1	98 ± 2	100 ± 3	74 ± 2	0.8	–24.5
14	120 ± 3	96 ± 2	101 ± 2	99 ± 3	–16.1	2.6

To determine the possible influence of the applied magnetic field on the crystal lattice parameters, crystals grown under the same solution supersaturation of 1.78%, without and in a magnetic field of 55 ± 3 mT were subjected to X-ray diffraction analyses at 293 K. The obtained data show that all of the observed crystals crystallized in a cubic system with space group *P*2_1_3. The crystal data and experimental details of the structure determination are given in Tables 4 and 5. The lattice parameters shown in the tables are *a*, the lattice constant; *V*, the cell volume; and *Z*, the number of molecules per unit cell.

**Table 4:** Data and Experimental Details for Crystals Grown in a Zero Magnetic Field

	crystal 1	crystal 2	crystal 3
Crystal Data			
chemical formula	ClNaO_3_	ClNaO_3_	ClNaO_3_
*M*_r_	106.44	106.44	106.44
cell setting, space group	cubic, *P*2_1_3	cubic, *P*2_1_3	cubic, *P*2_1_3
*a* (Å)	6.5629 (3)	6.5601 (3)	6.5760 (3)
*V* (Å^3^)	282.67 (4)	282.31 (4)	284.37 (4)
*Z*	4	4	4
no. of reflections for cell measurement	202	239	249
θ range (degree) for cell measurement	4.4–29.1	3.1–29.1	3.1–28.5
μ (mm^–1^)	1.26	1.26	1.25
crystal form, color	cube, colorless	cube, colorless	cube, colorless
crystal size (mm)	0.67 × 0.63 × 0.35	0.51 × 0.48 × 0.34	0.73 × 0.59 × 0.42
Data Collection			
absorption correction	multiscan	multiscan	multiscan
*T*_min_, *T*_max_	0.594, 1.000	0.620, 1.000	0.656, 1.000
reflections collected	248	280	281
independent reflections	186	168	195
observed reflections [*I* > 2σ(*I*)]	181	167	194
*R*_int_	0.016	0.010	0.011
θ values (degree)	θ_max_ = 29.2, θ_min_ = 4.4	θ_max_ = 29.4, θ_min_ = 4.4	θ_max_ = 29.1, θ_min_ = 4.4
range of *h*, *k*, *l*	*h* = −6 → 1, *k* = −3 → 8, *l* = −4 → 6	*h* = −4 → 8, *k* = −4 → 9, *l* = −4 → 4	*h* = −8 → 3, *k* = −5 → 5, *l* = −1 → 6
Refinement			
*R*[*F*^2^ > 2σ(*F*^2^)], *w*R**(*F*^2^), *S*	0.024, 0.058, 1.16	0.030, 0.073, 1.27	0.031, 0.075, 1.30
no. of reflections	186	168	195
no. of parameters	17	17	17
no. of restraints	0	0	0
weighting scheme	*w* = 1/[σ^2^(*F*_o_^2^) + (0.0333*P*)^2^], where *P* = (*F*_o_^2^ + 2*F*_c_^2^)/3	*w* = 1/[σ^2^(*F*_o_^2^) + (0.0485*P*)^2^], where *P* = (*F*_o_^2^ + 2*F*_c_^2^)/3	w = 1/[σ^2^(*F*_o_^2^) + (0.0473*P*)^2^ + 0.0113*P*], where *P* = (*F*_o_^2^ + 2*F*_c_^2^)/3
Δρ_max_, Δρ_min_(e Å^–3^)	0.31, −0.40	0.36, −0.55	0.47, −0.53

**Table 5:** Data and Experimental Details for Crystals Grown in an Applied Magnetic Field of 55 ± 3 mT

	crystal 1	crystal 2	crystal 3
Crystal Data			
chemical formula	ClNaO_3_	ClNaO_3_	ClNaO_3_
*M*_r_	106.44	106.44	106.44
cell setting, space group	cubic, *P*2_1_3	cubic, *P*2_1_3	cubic, *P*2_1_3
*a* (Å)	6.5666 (3)	6.5788 (6)	6.5686 (3)
*V* (Å^3^)	283.15 (4)	284.73 (8)	283.41 (4)
*Z*	4	4	4
no. of reflections for cell measurement	214	173	210
θ range (degree) for cell measurement	5.4–29.1	3.2–29.3	3.1–28.5
μ (mm^–1^)	1.26	1.25	1.26
crystal form, color	cube, colorless	cube, colorless	cube, colorless
crystal size (mm)	0.66 × 0.53 × 0.32	0.61 × 0.50 × 0.46	0.58 × 0.54 × 0.32
Data Collection			
absorption correction	multiscan	multiscan	multiscan
*T*_min_, *T*_max_	0.630, 1.000	0.625, 1.000	0.626, 1.000
reflections collected	270	264	264
independent reflections	195	185	189
observed reflections [*I* > 2σ(*I*)]	193	182	185
*R*_int_	0.035	0.015	0.016
θ values (degree)	θ_max_ = 28.6, θ_min_ = 4.4	θ_max_ = 28.3, θ_min_ = 4.4	θ_max_ = 28.5, θ_min_ = 4.4
range of *h*, *k*, *l*	*h* = −8 → 4, *k* = −7 → 2, *l* = −4 → 5	*h* = −5 → 4, *k* = −4 → 8, *l* = −3 → 8	*h* = −4 → 8, *k* = −8 → 3, *l* = −5 → 4
Refinement			
*R*[*F*^2^ > 2σ(*F*^2^)], *w*R**(*F*^2^), *S*	0.046, 0.103, 1.25	0.031, 0.084, 1.09	0.031, 0.071, 1.08
no. of reflections	195	185	189
no. of parameters	17	17	17
no. of restraints	0	0	0
weighting scheme	*w* = 1/[σ^2^(*F*_o_^2^) + (0.0695*P*)^2^] where *P* = (*F*_o_^2^ + 2*F*_c_^2^)/3	*w* = 1/[σ^2^(*F*_o_^2^) + (0.0605*P*)^2^] where *P* = (*F*_o_^2^ + 2*F*_c_^2^)/3	*w* = 1/[σ^2^(*F*_o_^2^) + (0.0461*P*)^2^] where *P* = (*F*_o_^2^ + 2*F*_c_^2^)/3
Δρ_max_, Δρ_min_(e Å^–3^)	0.57, −0.94	0.35, −0.30	0.35, −0.46

For a brief overview, Table 6 shows the values of the crystal parameters obtained by X-ray analyses.

**Table 6:** Lattice Parameters

zero field			magnetic field of 55 ± 3 mT		
*n*	*a* (Å)	*V* (Å^3^)	*n*	*a* (Å)	*V* (Å^3^)
1	α = β = γ = 90°, 6.5629(3)	282.67(4)	1	α = β = γ = 90°, 6.5666(3)	283.15(4)
2	α = β = γ = 90°, 6.5601(3)	282.31(4)	2	α = β = γ = 90°, 6.5788(6)	284.73(8)
3	α = β = γ = 90°, 6.5760 (3)	284.37(4)	3	α = β = γ = 90°, 6.5686(3)	283.41(4)

## Discussion

From the results presented in Table 1 and the scatter diagram in Figure 3, it is can be noted that sodium chlorate crystals nucleated and grown in a magnetic field of 55 ± 3 mT, in the studied supersaturation range of 0.89–1.78%, grew at slightly higher rates in the ⟨100⟩ direction than crystals grown under zero field conditions. It was found that the relative changes in the mean growth rates as a result of the influence of the magnetic field were 14.2, 7.7, 8.7, 10.9, and 12.6%, for the supersaturations listed in Table 1.

### Thermal Effect of the Magnetic Field

The increase in mean growth rates caused by the magnetic field is equivalent to the effect of increase in the saturation temperature of the solution. It was recently shown that the {100} faces of sodium chlorate crystals can grow by different mechanisms in the supersaturation range of 0.44–1.32%.^17^ The functions describing the spiral growth were found to be practically linear for supersaturations higher than 0.89% (Figure 2).^17^ Negative values of the growth rate have no physical meaning, but in theories of spiral growth, the intercept of the linear function with the abscissa has the meaning of critical supersaturation, σ_c_. Its estimated value for the experiments presented is 0.19%. According to theories of spiral growth, a linear (*R*, σ) dependence appears for supersaturations σ ≫ σ_c_. The used supersaturations of 0.89–1.78% satisfy this criterion. For this reason and for simplicity, a linear function was used to estimate the thermal effect of the magnetic field, i.e., the value of the saturation temperature change. A fit of the (*R*, σ) dependence for zero field conditions to a linear function was performed. By combining the obtained empirical equation (*R* = −9.94 + 52.83σ) with the formula for concentration,^10^ the values for the temperature change and the corresponding supersaturation of the solution were calculated and are presented in Table 7. The parameters *T*_0_′ and σ′ represent the calculated saturation temperature and relative supersaturation of the solution which would cause the same increase in the crystal growth rate as the applied magnetic field.

**Table 7:** Measured and Calculated Parameters of the Solution

*T*_0_ [°C]	σ [%]	*T*_0_′ [°C]	σ′ [%]	Δ*T*_0_′ [°C]
30.0	0.89	30.2	0.96	0.2
30.5	1.11	30.7	1.20	0.2
31.0	1.34	31.3	1.46	0.3
31.5	1.56	31.8	1.70	0.3
32.0	1.78	32.4	1.97	0.4

It could be assumed that the applied magnetic field would have an effect on the observed ⟨100⟩ mean growth rates of the sodium chlorate crystals, which correspond to an increase in the saturation temperature of the solution Δ*T*_0_′.

To confirm this assumption, experiments were performed in which 34 crystals were nucleated and grown at 28.0 ± 0.1 °C from a solution saturated at 32.4 ± 0.1 °C. The ⟨100⟩ mean growth rate of these crystals was 96 ± 7 nm s^–1^. Considering experimental errors, it is in good agreement with mean growth rate of crystals 94 ± 4 nm s^–1^ grown in a magnetic field from solutions saturated at 32 °C. This is in agreement with the assumption that the applied magnetic field has an effect corresponding to an increase in the saturation temperature of the solution, based on the empirical equation *R* = −9.94 + 52.83σ.

### Thermodynamic Effect

To determine whether the thermodynamic effect of the applied magnetic field can contribute to the increase of growth rates of the observed crystals, the temperature shift Δ*T* (the corresponding isothermal change of supersaturation) was estimated. The proposed formula was used,^3^, where *H* is the strength of the magnetic field, *T* is the growth temperature, Δ_0_*H* is the molar enthalpy of crystallization, μ_0_ is the magnetic permeability of the vacuum, and χ is the molar magnetic susceptibility of the solution. The estimated value of the temperature shift of the crystallization temperature caused by the magnetic field is Δ*T* ≈ 4.2 × 10^–4^. The positive value of Δ*T* is characteristic of diamagnetic substances, such as sodium chlorate, and applied magnetic field shifts the equilibrium temperature to a higher value for diamagnetic crystals. However, the value of Δ*T* is too small, and it can be concluded that the thermodynamic effect is not responsible for the increase in the ⟨100⟩ mean growth rates of the observed sodium chlorate crystals grown in the external magnetic field.

### Magnetohydrodynamic Effect

This effect is based on the concept that the Lorentz force acts on ions moving in a magnetic field, changing the direction of their flow. Considering this effect in a very simplified way, it depends on the conductivity of the ionic solution, the velocity of ion movement in it, the strength of the applied magnetic field, and the magnetic permeability of the crystals. In the experiments described in this paper, the nucleation and growth of diamagnetic crystals were observed in an ionic solution with a conductivity of less than 200 mS cm^–1^ (measured with the WTW Cond 330i). The solution rate at the bottom of the cell, which can be considered the velocity of the ion current, was about 0.05 mm s^–1^, and the magnetic permeability was ≈1. Under these experimental conditions, there was a slight increase in the growth rates of the observed crystals (Figure 3 and Table 1). Schieber^1^ found no effect of the magnetic field on the growth rates of the diamagnetic KAl(SO_4_)_2_·12H_2_O crystals in his experiments, while the increase in the growth rates of the paramagnetic Fe(NH_4_)_2_(SO_4_)_2_·6H_2_O was measurable. Since the magnetic permeability was ≈1 for both para- and diamagnetic samples, and the expected field effect was not apparent for either type of studied crystals, he concluded that the magnetohydrodynamic effect cannot be a mechanism causing changes in the growth rates of the observed crystals. The results presented in this article give a possibility that this effect could be a mechanism responsible for increasing the ⟨100⟩ growth rates of sodium chlorate crystals. If this effect exists, there is no way to measure it, so it cannot be ruled out as a possible cause.

The magnetic dipolar interaction, the gradient of the magnetic field, and the wave mechanism of the external field effect depend on the orientation of the crystal to the field.^1,9^

The magnetic dipolar interaction, which has been shown to deform the shape of diffusion layers, should cause an increase in the growth rate of crystals with suitable orientation to the external magnetic field.^1^

The gradient of the magnetic field causes the appearance of an ion current that hinders the attachment of growth units to the crystal faces parallel to the field, i.e., reduces the rate in the direction perpendicular to the field.^1^

The wave mechanism of the effect of external fields on crystallization is based on the assumption that thermal vibrations of growth units in aqueous solution produce electrostatic waves.^9^ The growth units are separated by the electrostatic field of the generated waves, and their deposition rate is determined by the ratio between the frequency of the electrostatic waves and the oscillation frequency of the units in the crystal lattice. The ratio of these frequencies determines the growth rates of the crystal. The introduction of an additional field would cause anisotropy of the crystal growth rates in orthogonal directions.^9^

The crystals observed in the experiments described were spontaneously nucleated, and there is no preferred orientation of the crystal nuclei to the applied magnetic field, which is illustrated in Figure 2. It shows that the majority of the observed crystals grew at rates close to the mean growth rate for a given supersaturation (illustrated in Figure 4) and that there were crystals with different orientations to the field that grew at high rates. It can be concluded that the growth rates of the crystals in the ⟨100⟩ direction are not significantly dependent on the angle between the magnetic field and the ⟨100⟩ direction. However, it can be noted that crystals oriented on angles between 40 and 60° grew at highest rates. Previous studies suggested that a magnetic field should increase growth rates in the direction parallel to the field.^1^ The results presented in Table 2 show that the potential influence of the magnetic field on ⟨100⟩ crystal growth rates, orthogonal or parallel to the field, is small. This is confirmed by the results shown in Table 3. Also, the results from Table 2, and maybe Figure 4 suggest that the greatest effect of the field is on crystals oriented at 40–60°, which is in agreement with previous results.^1^

Obtained results suggest that magnetic dipolar interaction and magnetic field gradient are not responsible for the higher ⟨100⟩ mean growth rates of the observed sodium chlorate crystals nucleated and grown in the magnetic field of 55 ± 3 mT, while the influence of wave mechanism cannot be excluded.

The results of X-ray analysis presented in Tables 4–6 show that two of the three analyzed crystals nucleated and grown in a magnetic field of 55 ± 3 mT have a slightly higher lattice constant than crystals grown at the same solution supersaturation of 1.78%, but in zero field. The average change in lattice constant of these two crystals caused by this weak magnetic field is 0.15%.

Although two of three crystals grown in a magnetic field had higher crystal lattice parameters than crystals grown without the field, further research is needed for possible conclusions about the effect of the field on the crystal lattice parameters. The possible influence of the magnetic field on the lattice parameters perhaps can be analogous to the influence of temperature on the misorientation of the mosaic blocks and the mosaic spread,^18^ which affect the crystal growth rate.^19^ If the lattice parameters are higher in the magnetic field, this can lead to a decrease in the mosaic spread, like it is caused by an increase in temperature,^18^ which can be the cause of a higher rate of crystal growth in the magnetic field.^19^

## Conclusions

Herein, the results of the research on the influence of the magnetic field on the growth of diamagnetic sodium chlorate crystals are presented. Based on the presented results, the following conclusions can be drawn:

1. The applied magnetic field of 55 ± 3 mT slightly increases the ⟨100⟩ mean growth rates of the sodium chlorate crystals in the supersaturation range of 0.89–1.78%. An average estimated relative change in the mean growth rate is 10.8%.
2. The magnetic field has a thermal effect corresponding to the relative increase in the saturation temperature of the solution for values of 0.55, 0.62, 0.92, 1.03, and 1.29%, corresponding to the solution supersaturations of 0.96, 1.20, 1.46, 1.70, and 1.97%.
3. The thermodynamic effect of the magnetic field cannot be responsible for the increase in the ⟨100⟩ mean growth rates of observed sodium chlorate crystals. The estimated value of the temperature shift is Δ*T* ≈ 4.2 × 10^–4^ K, too small to cause any effect.
4. The magnetohydrodynamic effect could be a mechanism leading to an increase in the ⟨100⟩ growth rate of sodium chlorate crystals. However, it must be taken into account that nucleation and growth of the crystals occurred in a solution with conductivity less than 200 mS cm^–1^, an ionic current velocity of about 0.05 mm s^–1^, and a magnetic permeability of diamagnetic samples ≈ 1, without the possibility of measuring the magnetohydrodynamic effect itself.
5. The magnetic dipolar interaction and the magnetic field gradient are not responsible for higher growth rates of sodium chlorate crystals observed in a magnetic field, while the influence of the wave mechanism cannot be excluded.
6. X-ray analyses indicate that crystals nucleated and grown in the magnetic field of 55 ± 3 mT might have a slightly higher lattice parameter. Namely, two of three analyzed crystals, nucleated and grown in a field, had slightly higher lattice constant. Regardless of this fact, additional analyses are needed to draw clear conclusions.

The application of a certain value of magnetic field could lead to products with certain properties. However, the results of the presented study cannot yet provide complete information leading to a full understanding of the phenomenon of the influence of the magnetic field on the growth of sodium chlorate crystals.

## Supporting Information Available

Single-crystal diffraction analysis of sodium chlorate crystals grown from aqueous solution at a supersaturation of 1.78%
Crystallographic data of crystal 1 grown in a magnetic field of 55 ± 3 mT (CIF)
Crystallographic data of crystal 1 grown in zero field conditions (CIF)
Crystallographic data of crystal 2 grown in a magnetic field of 55 ± 3 mT (CIF)
Crystallographic data of crystal 2 grown in zero field conditions (CIF)
Crystallographic data of crystal 3 grown in a magnetic field of 55 ± 3 mT (CIF)
Crystallographic data of crystal 3 grown in zero field conditions (CIF)

## Supplementary Material

ao2c04790_si_001.cif

ao2c04790_si_002.cif

ao2c04790_si_003.cif

ao2c04790_si_004.cif

ao2c04790_si_005.cif

ao2c04790_si_006.cif
